# Bisphosphonate-induced orbital inflammation in a patient on chronic immunosuppressive therapy

**DOI:** 10.1186/s12886-019-1063-8

**Published:** 2019-02-14

**Authors:** Isabella Herrera, Yong Kam, Thomas J. Whittaker, Mary Champion, Radwan S. Ajlan

**Affiliations:** Department of Ophthalmology, University of Kansas School of Medicine, 7400 State Line Rd, Prairie Village, Kansas, USA

**Keywords:** Orbital inflammation, Orbital pseudotumor, Bisphosphonate therapy, Immunosuppression

## Abstract

**Background:**

To report a case of orbital inflammation after bisphosphonate infusion in a patient who was already receiving immunosuppressive therapy.

**Case presentation:**

A 56-year-old woman presented to the ophthalmology clinic with acute onset of right eye pain 24 h after receiving her first Zolendronic acid infusion. She has a past medical history of chronic inflammatory demyelinating polyneuropathy, Sjogren’s syndrome, and systemic lupus erythematosus that have been controlled with immunosuppressive therapy for three years. Clinical ophthalmic exam and MRI studies were significant for right orbital inflammation. The patient was started on oral prednisone with rapid resolution of symptoms.

**Conclusions:**

This is the first case report of a patient receiving chronic immunosuppressive therapy to develop orbital inflammation after Zoledronic acid infusion. In addition, it demonstrates that corticosteroids can be an effective first line therapy in treating orbital inflammation in similar patients. Physicians should be aware of this rare but serious potential side effect of bisphosphonates, and have bisphosphonate-related orbital inflammation on their differential for proper initiation of treatment.

## Background

Bisphosphonates are widely prescribed and are highly effective in the treatment of osteoporosis, Paget’s disease, calcium disorders, and certain metastatic bone disease. Zoledronic acid is an intravenous (IV) bisphosphonate, which can be used in patients with intolerance to oral bisphosphonates. The most commonly associated side effects are flu-like symptoms (nausea, fever, and myalgia) that occur within days after administration [1]. Ocular complications are less frequent, and can manifest as anterior uveitis, scleritis, episcleritis, conjunctivitis, and/or orbital inflammation [2]. There are few reported cases of orbital inflammation following bisphosphonate treatment [3]. To the best of our knowledge, this is the first case report in the literature of orbital inflammation after zoledronic acid infusion in a patient on systemic immunosuppressive therapy.

## Case presentation

A 56-year-old woman presented to the ophthalmology clinic with a main complaint of acute onset right eye pain with extra-ocular movement. She also reported right eye periorbital swelling, redness, upper right lid drooping, and bilateral tearing. She received her first zoledronic acid infusion for osteoporosis 24 h prior to presentation. Her significant past medical history included chronic inflammatory demyelinating polyneuropathy, Sjogren’s syndrome, and systemic lupus erythematosus. The patient had been on cyclosporine 75 mg (1 mg/kg) daily and monthly belimumab 120 mg/1.5 ml for her rheumatologic conditions for 3 years prior to presentation.

**Fig. 1:**
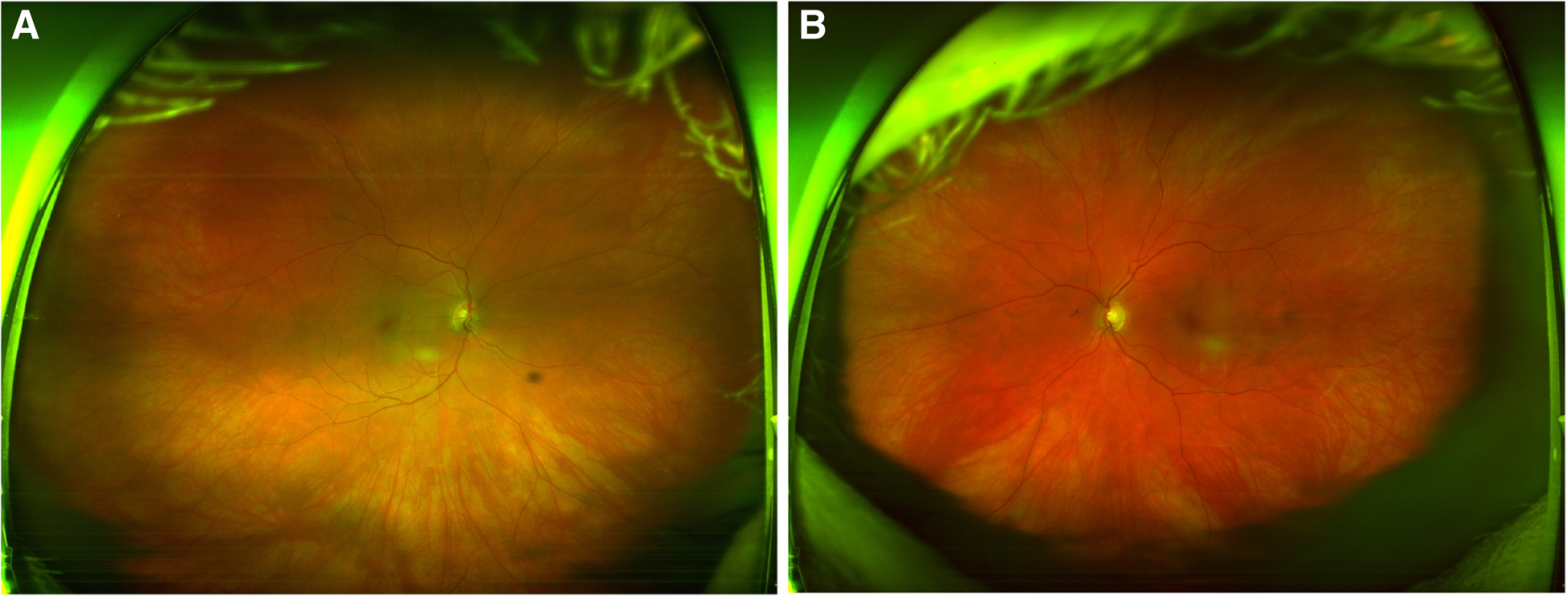
Ultra-wide field fundus photos of both eyes (**a**: right eye, **b**: left eye). In addition to floaters in both eyes, both optic nerve heads appear sharp with no disc edema

**Fig. 2:**
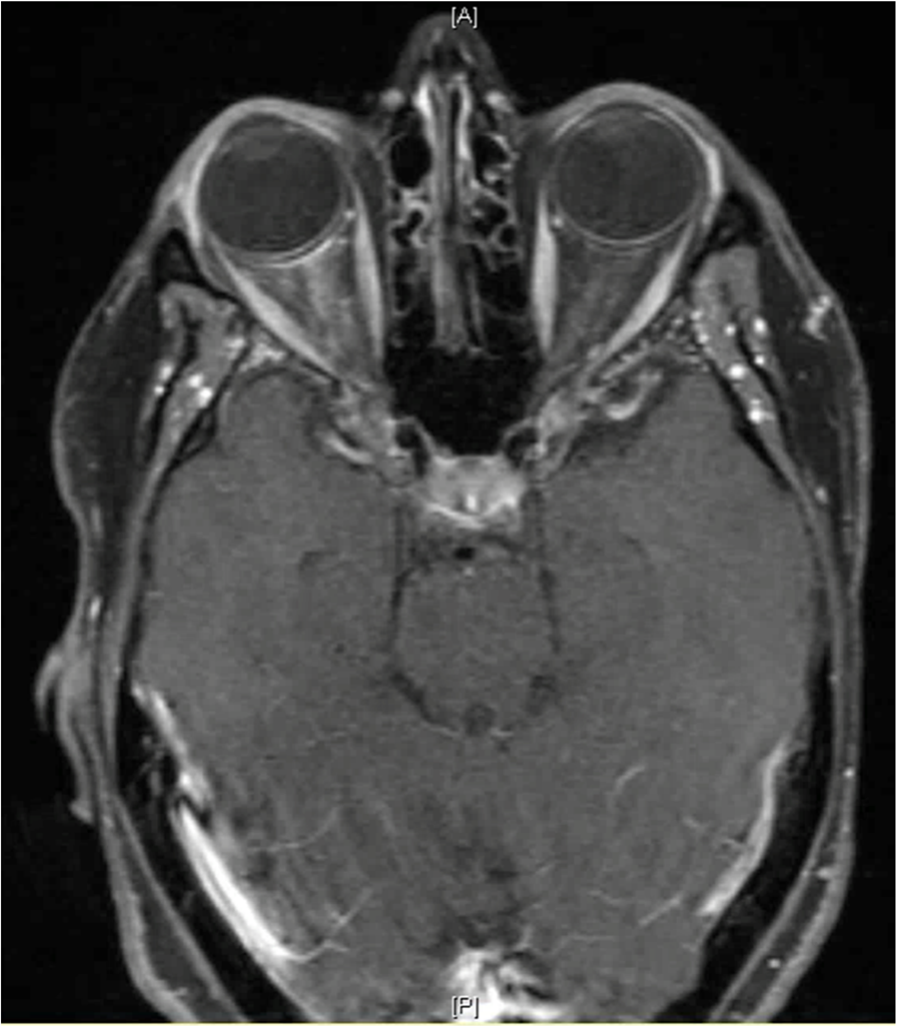
Axial T1 weighted fat suppression post contrast MRI showing right intraconal fat and posterior scleral enhancement

**Fig. 3:**
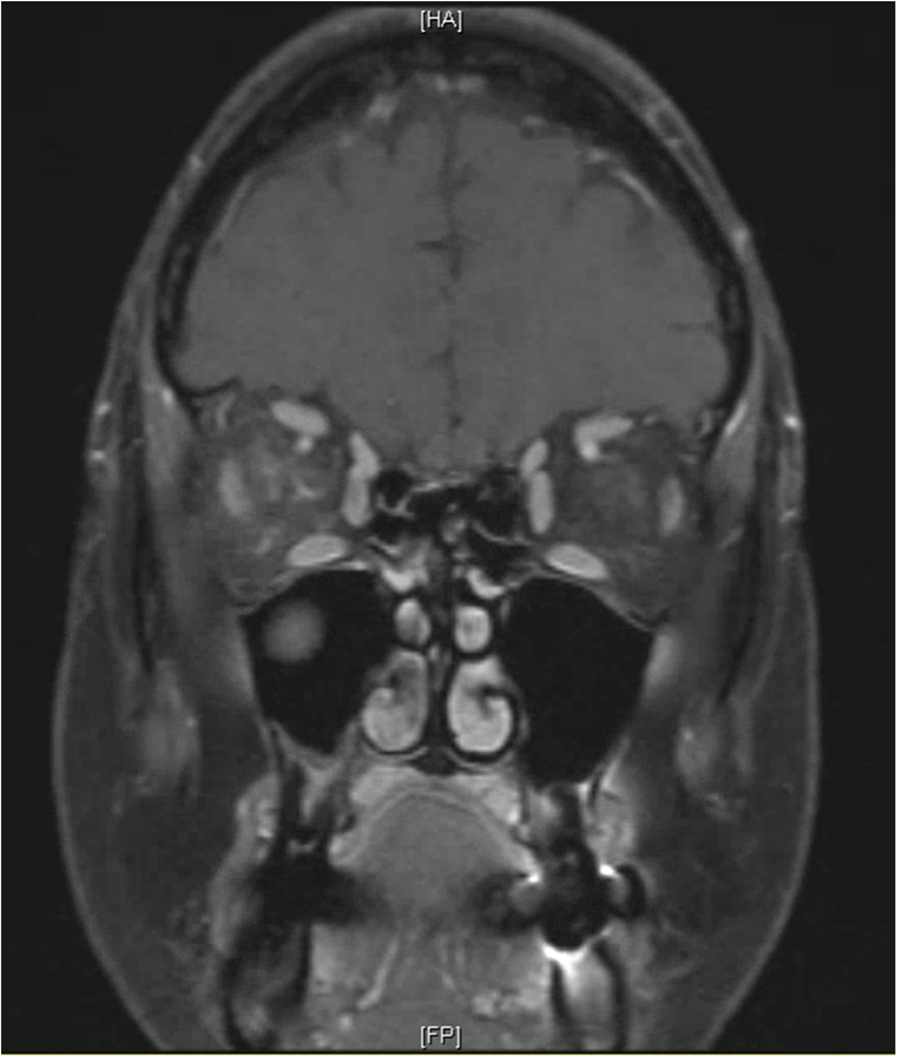
Coronal T1 weighted fat suppression post contrast MRI showing right intraconal fat and optic nerve sheath enhancement without optic nerve enhancement

On ophthalmic exam, her best-corrected visual acuity was 20/25–2 in the right eye, and 20/25–3 in the left eye. Intraocular pressure was 14 mmHg in the right eye and 13 mmHg in the left eye. On external exam of the right eye, there was mild upper lid edema, erythema, ptosis, and − 1 adduction defect. The slit lamp exam was remarkable for conjunctival chemosis without anterior chamber cell or flare. The posterior segment exam was remarkable for posterior vitreous detachment (PVD) (Fig. 1). The left eye exam was significant for PVD (Fig. 1). 24–2 visual field testing attempted but was not reliable due to frequent fixation loss secondary to eye pain. A contrast enhanced MRI was obtained which showed: 1) ill-defined right orbital soft tissue thickening, 2) enhancement in the retro-orbital intraconal space with extension along the retro-orbital scleral contour and surrounding the anterior optic nerve sheath (Figs. 2 and 3). Orbital inflammation secondary to SLE was considered as part of the differential based on the patient’s rheumatologic history. However, based on the clinical presentation, MRI findings, and timing of symptoms with regards to the zoledronic acid infusion, orbital inflammation secondary to bisphosphonate therapy was suspected. The patient was started on oral prednisone 50 mg daily with rapid improvement in symptoms. After 1 week, patient reported full resolution of symptoms and was started on a slow weekly taper of prednisone.

## Discussion and conclusions

This case illustrates a rare complication of orbital inflammation after zoledronic acid treatment in a patient who is already on chronic immunosuppression. The mechanism for this adverse effect has been hypothesized to be associated with the acute inflammatory response from the activation of T-cells and cytokine release [4]. Patients with ophthalmic involvement typically report having flu-like symptoms lasting for 24 to 72 h prior to onset of orbital disease^2^. Depending on the route of bisphosphonate administration, ophthalmic symptoms can present within three days after IV bisphosphonate infusion or within 2–3 weeks after oral bisphosphonate treatment [1–3, 5, 6]. Signs and symptoms include decreased vision, periorbital edema and erythema, ptosis, painful and restricted eye movements, proptosis, conjunctival injection and chemosis, posterior scleritis, anterior uveitis, and optic nerve edema [5]. The relatively mild presenting symptoms in this case may be explained by the T-cell inhibition effect of cyclosporine therapy, and possible B-cell activation inhibition effect by belimumab on T-cell activation and/or recruitment. Imaging with MRI or CT may reveal preseptal or orbital fat inflammation, extraocular muscle enlargement, lacrimal gland inflammation, scleral thickening and optic nerve abnormality [1–3, 5]. Extraocular muscles are rarely involved, but when affected they tend to be minimally enlarged [2, 6]. Steroids have been reported as first line therapy for orbital inflammation secondary to bisphosphonates in several cases in literature, with complete resolution of symptoms shortly after treatment. Low-dose radiation therapy is used when there is a contraindication or poor response to steroids, or there is a recurrence of disease with tapering of steroid. If symptoms progress with steroids therapy, an infectious etiology should be evaluated. Orbital inflammation has been hypothesized to be immune-mediated, and thus cyclosporine, chlorambucil, and indomethacin have been used as alternative treatments in refractory cases [7–9].

This is the first reported case of a patient already on chronic immunosuppressive therapy to develop orbital inflammation after zolendronic acid infusion. Nevertheless, this case report illustrates that even patients on immunosuppressive therapy are susceptible to developing this complication of bisphosphonate therapy. The rapid resolution of symptoms supports the use of high-dose corticosteroids as first line to treat orbital inflammation in patients on chronic immunosuppression. Physicians should be aware of this rare but serious potential side effect of bisphosphonates.

## Abbreviations

CT: Computed tomography
MRI: Magnetic resonance imaging
PVD: Posterior vitreous detachment
SLE: Systemic lupus erythematosus

## References

[1] Manuylova E, Clark N, Shafiq I (2016). Orbital Pseudotumor in a patient treated with Zoledronic Acd: a case report and pertinent literature review. AACE Clinical Case Reports.

[2] Rahimy E, Law SK (2013). Orbital inflammation after zoledronate infusion: an emerging complication. Can J Ophthalmol.

[3] Miguel GBJ, Toman OB, Guillermo HG (2016). Orbital inflammation caused by bisphosphonates case report and literature review. Int J Ocul Oncol Oculoplasty.

[4] Rossini M, Adami S, Viapiana O (2013). Acute phase response after zoledronic acid is associated with long-term effects on white blood cells. Calcif Tissue Int.

[5] Pirbhai R, Rajak SN, Goold LA (2015). Bisphosphonate-induced orbital inflammation: a case series and review. Orbit.

[6] Lefebre DR, Mandeville JT, Tonekawa Y (2016). A case series and review of bisphosphonate-associated orbital inflammation. Ocul Immunol Inflamm.

[7] Jacobs D, Galetta S (2002). Diagnosis and management of orbital pseduotumor. Curr Opin Ophthalmol.

[8] Diaz-Llopis M, Menezo JL (1989). Idiopathic inflammatory orbital pseudotumor and low- dose cyclosporine. Am J Ophthalmol.

[9] Zacharopoulos IP (2009). Treatment of idiopathic orbital inflammatory disease with cyclosporine-a: a case presentation. Semin Ophthalmol.

